# Supplementary material for: Yoked surface codes
Source: Nat Commun. 2025 May 14;16:4498. doi: 10.1038/s41467-025-59714-1 (PMC12078507; doi:10.1038/s41467-025-59714-1)
Supplement: Supplementary file 1 — Supplementary Information [file 41467_2025_59714_MOESM1_ESM.pdf]

# Supplementary Information for Yoked Surface Codes


Craig Gidney,[1] Michael Newman,[1] Peter Brooks,[1] and Cody Jones[1]

[1] *Google Quantum AI, Santa Barbara, California 93117, USA*



(Dated: May 1, 2025)


## 1. SUPPLEMENTARY NOTE 1 – OVERHEAD ESTIMATES WITH STANDARD DEPOLARIZING NOISE

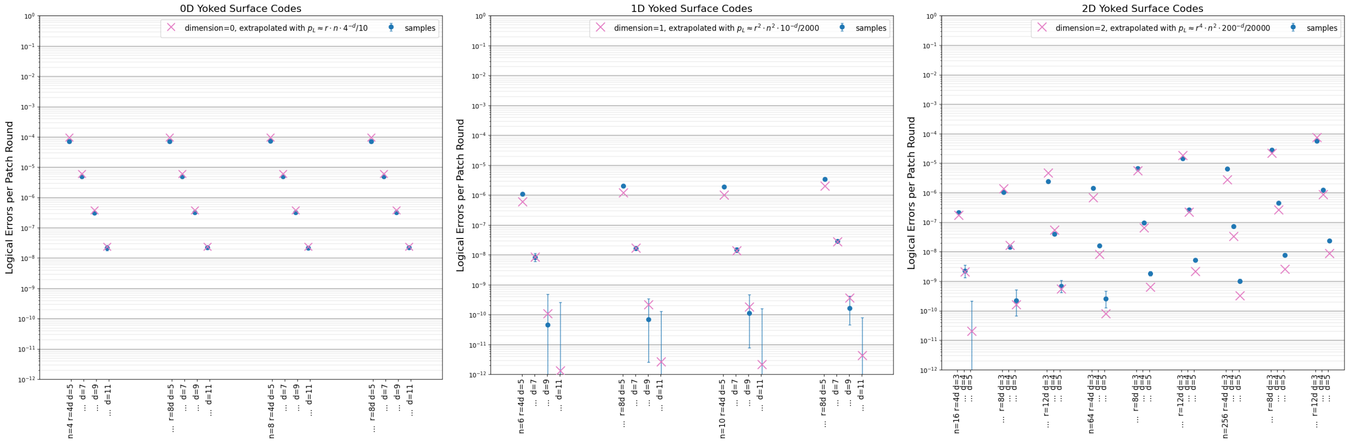


Supplementary Figure 1: **Gap simulations using uniform circuit-level depolarizing noise**. Left: 0D (i.e. normal surface codes), middle: 1D, and right: 2D yoked surface codes. Error bars represent hypothetical logical error rates with a Bayes factor of at most 1000 versus the maximum likelihood hypothesis probability, assuming a binomial distribution. Note that for 1D yoked surface codes, we did not sample any failures at inner code distance 11. However, as expected, the error bars cover the extrapolations denoted in the legend.

In the main text, we have used SI1000 noise to focus on superconducting qubit circuits. Compared to more standard uniform circuit-level depolarizing noise, these circuits tend to be noisier given the same error rate parameter $p$ [1]. The reason is that several of the operations are scaled to be noisier, reflecting noisier superconducting operations such as measurement - see Appendix 2.

To provide a point of comparison to more standard error models in the literature, we perform an abbreviated analysis using uniform circuit-level depolarizing noise. The exact error model and allowed operations are also described in Appendix 2. The analysis is similar to the main text, but using uniform depolarizing noise (again with $10^9$ samples per code distance and $p = 10^{-3}$) to generate the gap distributions. We run the least expensive studies - small gap simulations - to fit the scaling approximations, and use these to estimate overheads. In this more favorable noise model, we find the single-significant-figure fits

$$p_{L,0} \approx r_i \cdot n \cdot 4^{-d}/10$$
$$p_{L,1} \approx r_o \cdot r_i^2 \cdot n^2 \cdot 10^{-d}/2000$$
$$p_{L,2} \approx r_o \cdot r_i^4 \cdot n^2 \cdot 200^{-d}/20000,$$

where again $p_{L,k}$ denotes the cumulative logical error rate of $k$-D yoked surface codes over $r_o$ rounds of the outer code, with $r_i$ rounds between checks, for size-$n$ code blocks, and distance-$d$ inner surface codes. We note that the 1D yoked surface code fit is especially conservative - considering Supplementary Figure 1, the error suppression factor likely lies somewhere between 10 and 20. However, full circuit simulations are needed to pin down these numbers past single-significant-figure estimates. In Supplementary Figure 2, we estimate that 2D yoked surface codes can preserve a logical information for up to $\sim 10^{12}$ logical-qubit-rounds using about 350 physical qubits per logical qubit (compared to 500 physical qubits per logical qubit under SI1000 noise).

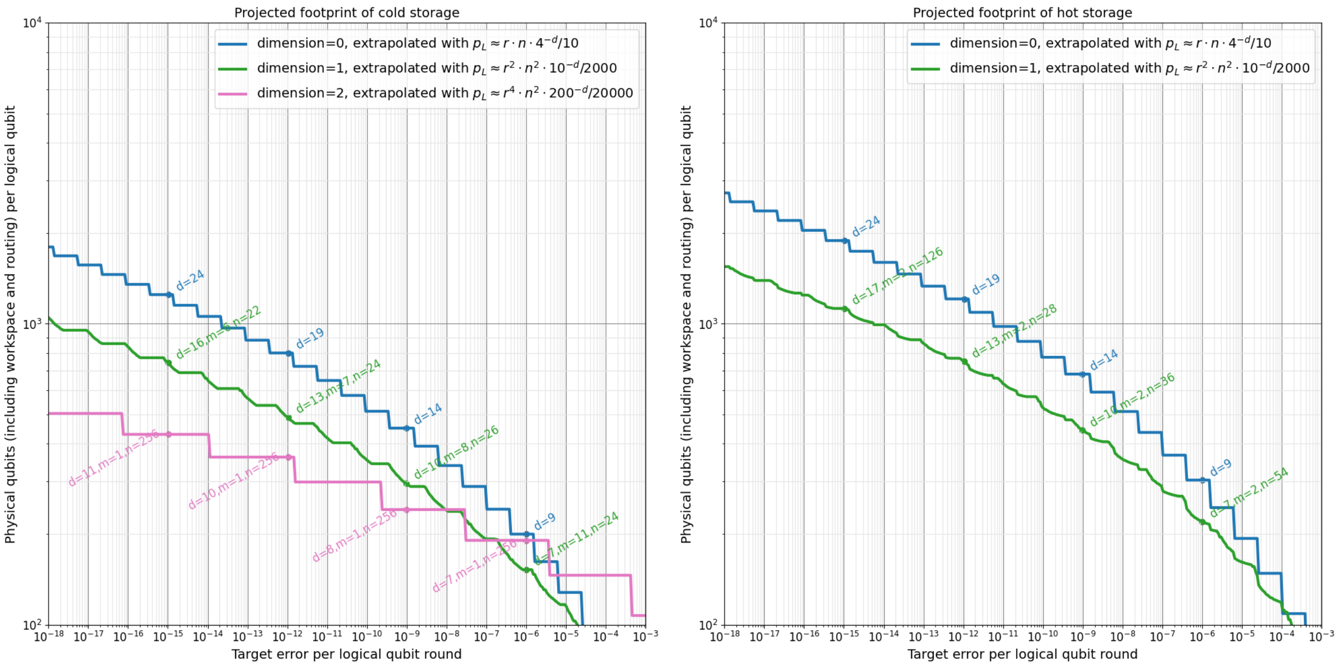


Supplementary Figure 2: **Extrapolated footprints using uniform circuit-level depolarizing noise.** These include 0D (standard), 1D, and 2D yoked surface codes and assume a physical error rate of $10^{-3}$. Projections for cold storage are on the left, and projections for hot storage are on the right. A patch of diameter $d$ is assumed to cover $2(d+1)^2$ physical qubits to leave some buffer space for lattice surgery. The yoked hot storage estimates target an access hallway utilization of 40%. 2D yoked surface codes can nearly achieve a $10^{-12}$ error per logical-qubit-round using about 350 physical qubits per logical qubit.

## 2. SUPPLEMENTARY NOTE 2 – NOISE MODELS

Simulations in the main text were done using the superconducting-inspired circuit noise model defined in Supplementary Table 1. The name "SI1000" is short for Superconducting Inspired with 1000 nanosecond cycle.

In Appendix 1, we study a more standard uniform circuit-level noise model similar to that used e.g. in [2, 3]. This error model is described in Supplementary Table 2.

| Ideal gate | Noisy replacement |
|---|---|
| (any single qubit unitary, including idling) $U_1$ | $\mathrm{DEP1}(p/10) \cdot U_1$ |
| CZ | $\mathrm{DEP2}(p) \cdot \mathrm{CZ}$ |
| $R_Z$ | $\mathrm{XERR}(2p) \cdot R_Z$ |
| $M_Z$ | $\mathrm{DEP1}(p) \cdot \mathrm{MERR}_Z(5p)$ |
| (Wait for $M_Z$ or $R_Z$) | $\mathrm{DEP1}(2p)$ |

Supplementary Table 1: **The superconducting-inspired noise model "SI1000" used by simulations in the main text.** The single parameter $p$ sets the two-qubit gate error rate, with other error rates defined relative to this rate. Measurements are noisiest while single qubit gates are least noisy. Qubits that are not reset or measured during layers containing resets or measurements incur additional depolarization on top of other error mechanisms. Noise channels are defined in Supplementary Table 3.

| Ideal gate | Noisy replacement |
|---|---|
| Idle | DEP1($p$) |
| (any single qubit Clifford)$U_1$ | DEP1($p$) $\cdot$ $U_1$ |
| (any two qubit Clifford)$U_2$ | DEP2($p$) $\cdot$ $U_2$ |
| $R_X$ | ZERR($p$) $\cdot$ $R_X$ |
| $R_Z$ | XERR($p$) $\cdot$ $R_Z$ |
| $M_X$ | DEP1($p$) $\cdot$ MERR$_X$($p$) |
| $M_Z$ | DEP1($p$) $\cdot$ MERR$_Z$($p$) |

Supplementary Table 2: **The uniform circuit-level depolarizing noise model used by simulations in Appendix 1.** Each qubit not actively participating in a gate experiences a noisy idle operation. Noise channels are defined in Supplementary Table 3.

| Noise channel | Probability distribution of effects | | | |
|---|---|---|---|---|
| MERR$_B$($p$) | $1-p \to M_B$<br>$p \to M_{(-1 \cdot B)}$ *(i.e. measurement result is inverted)* | | | |
| XERR($p$) | $1-p \to I$<br>$p \to X$ | | | |
| ZERR($p$) | $1-p \to I$<br>$p \to Z$ | | | |
| DEP1($p$) | $1-p \to I$<br>$p/3 \to X$<br>$p/3 \to Y$<br>$p/3 \to Z$ | | | |
| DEP2($p$) | $1-p \to I \otimes I$ | $p/15 \to I \otimes X$ | $p/15 \to I \otimes Y$ | $p/15 \to I \otimes Z$ |
| | $p/15 \to X \otimes I$ | $p/15 \to X \otimes X$ | $p/15 \to X \otimes Y$ | $p/15 \to X \otimes Z$ |
| | $p/15 \to Y \otimes I$ | $p/15 \to Y \otimes X$ | $p/15 \to Y \otimes Y$ | $p/15 \to Y \otimes Z$ |
| | $p/15 \to Z \otimes I$ | $p/15 \to Z \otimes X$ | $p/15 \to Z \otimes Y$ | $p/15 \to Z \otimes Z$ |

Supplementary Table 3: **Definitions of various noise channels.** Used by Supplementary Table 1 and Supplementary Table 2.

## 3. SUPPLEMENTARY NOTE 3 – Y-TYPE YOKES

For 1D yoked surface codes, we could also consider replacing the $X$- and $Z$-type yoke checks with a single $Y$-type yoke check. The reason is that the inner surface code qubits are highly biased: in a phenomenological noise model on a surface code of distance $d$, the minimum weight of a $Y$-type logical operator is $2d$. Although in a circuit-level error model the minimum weight of a $Y$-type error is again distance $d$ [4], the very specific alignment of errors causing this failure makes it relatively rare. In simulation, for physical error rates around $10^{-3}$, we observe that the surface code behaves like a code with effective distance $1.8d$ against $Y$-type errors - see Supplementary Figure 4.

This begs the question: why not use individual $Y$-type checks rather than joint $X$- and $Z$- type checks to increase the number of logical qubits per physical qubit? There are two reasons - first, while measuring the $Y$-type check, there is a point in the lattice surgery where we may have to increase the size of the surface code to ensure resilience to $Y$-type errors (see Supplementary Figure 3). Second, the potential savings are reduced by the need to access both the $X$- and $Z$-type boundaries of the surface code in order to perform the $Y$-type check. Consequently, upon initial consideration, it seems prudent to use standard $X$- and $Z$-type checks. However, taking advantage of the intrinsic bias of the surface code could prove profitable in future fault-tolerant constructions.

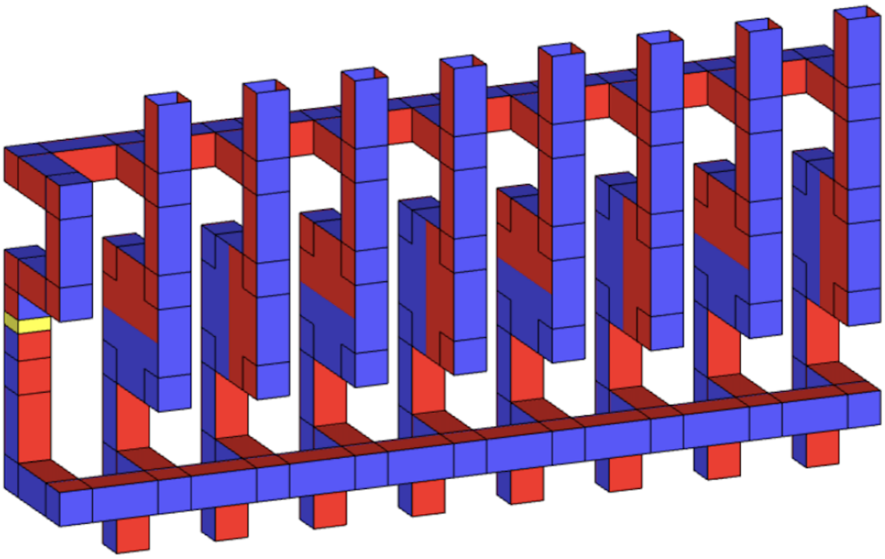

Supplementary Figure 3: **Lattice surgery construction for measuring a single $Y$-type check as an alternative 1D yoked surface code.** Assumes an even block size. The yellow block corresponds to a transversal Hadamard operation. Beware that this instantiation contains unsuppressed hook errors.

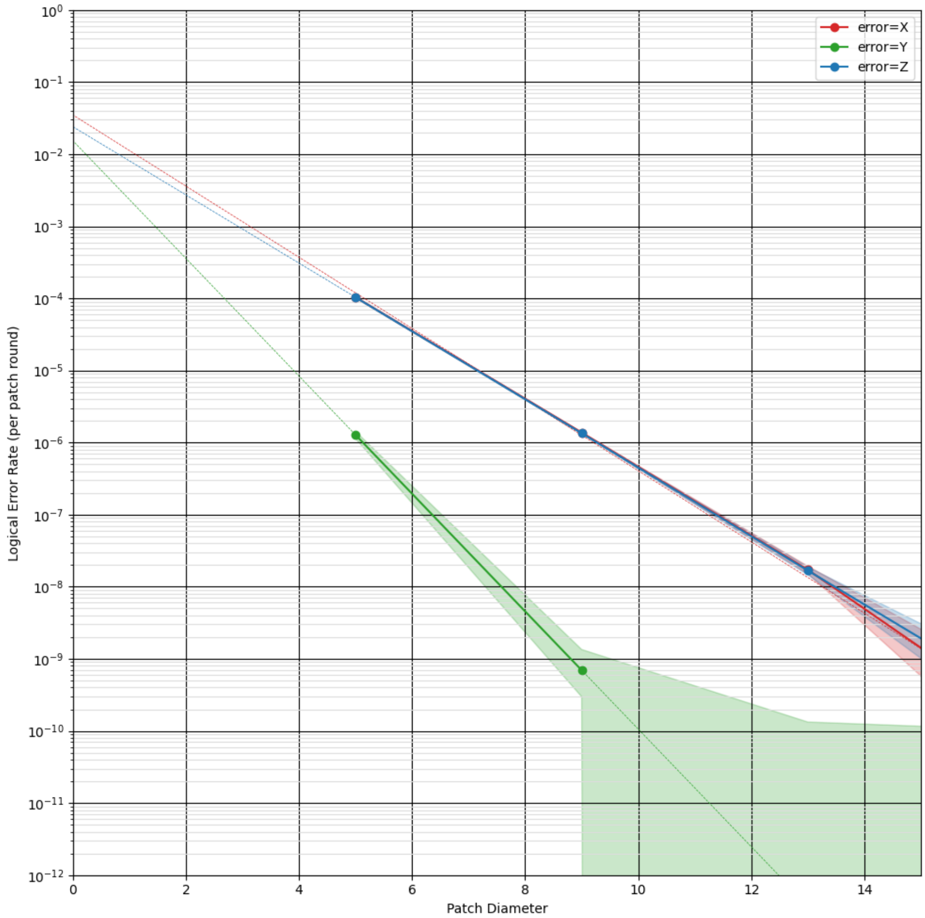


Supplementary Figure 4: **Logical X, Y, and Z error rates from a surface code memory circuit.** Logical errors are biased away from being $Y$-type logical errors. Empirically, the $Y$ error rates behave as if the code distance was $1.8 \cdot d$ (vs $1.0 \cdot d$ for $X$ and $Z$ errors), where $d$ is the patch diameter. Shading represents hypothetical logical error rates with a Bayes factor of at most 1000 versus the maximum likelihood hypothesis probability, assuming a binomial distribution.

## 4. SUPPLEMENTARY NOTE 4 – QUANTUM MULTI-DIMENSIONAL PARITY CHECK CODES

Classical multi-dimensional parity check codes (MDPCCs) are defined by arraying bits in an $r$-dimensional tensor. They are specified by a list of side lengths $(n_1, \dots, n_r)$. Each parity check is a row check along some dimension, i.e. a rank-$r$ delta tensor $\delta_{i_1,\dots,\hat{i_\ell},\dots,i_r}$, where $\hat{i_\ell}$ denotes a missing entry which is the free index - the degree of freedom whose parity is checked.

In 1D, this is simply a single parity check on all the bits. In 2D, this corresponds to laying out the bits in an $m \times n$ matrix, with $m$ row parity checks and $n$ column parity checks. For example, the $j$th column parity check would be denoted $\delta_{,j}$. The total number of bits is $n = \prod_i n_i$ and the minimum undetectable error is a configuration of bit flips forming the vertices of an $r$-dimensional rectangle within the array, and so $d = 2^r$.

To compute the number of encoded bits $k$, we can count the number of linearly independent parity checks. To do this, we iterate over the dimensions - in the $i_1$th direction, we have $n_2 n_3 \dots n_r$ parity checks (the area of the hyperface parallel to $i_1$). In the $i_2$th direction, we have $(n_1 - 1)n_3 n_4 \dots n_r$ independent parity checks, where $(n_1 - 1)$ accounts for the $n_3 n_4 \dots n_r$ parity checks that can be generated from the first $i_1$th direction parity checks and the $(n_1 - 1)n_3 n_4 \dots n_r$ parity checks in the $i_2$th direction. Continuing in this way, the $i_k$th direction contributes $(n_1 - 1)(n_2 - 1) \dots (n_{k-1} - 1)n_{k+1} \dots n_r$ independent parity checks. Expanding this polynomial representing $n - k$, we see that $k = \prod_{i=0}^{r} (n_i - 1)$.

To build quantum MDPCCs, we must construct both $X$-type and $Z$-type checks from this code. Unfortunately, simply assigning $X$- and $Z$-type generators from these parity checks directly won't yield a commuting set of stabilizers. Instead, when each $n_i \equiv (0 \mod 2^r)$, we can assign $X$-type generators to the parity checks defined by the MDPCC and $Z$-type generators to parity checks defined by a permutation of the MDPCC. In particular, because $n_i \equiv (0 \mod 2^r)$, we can write each parity check uniquely as

$$\delta_{i_1,\dots,\hat{i_\ell},\dots,i_r} = \delta_{s_1,\dots,\hat{i_\ell},\dots,s_n} \otimes \left( \bigotimes_{k=1}^{r} \delta_{b_{k_1},\dots,\hat{i_\ell},\dots,b_{k_r}} \right)$$

where each $\delta_{b_{k_1},\dots,\hat{i_\ell},\dots,b_{k_r}}$ is a $(2 \times \dots \times 2)$ rank-$r$ tensor. $X$- and $Z$-type stabilizers defined by these parity checks commute if and only if they have even parity when contracted along their ordered indices.

When two of these $(2 \times \dots \times 2)$-tensors differ in any index other than their free index, their contraction is zero. If they share the same free index, then their contraction must have even parity since the dimension of each index is two. The trouble comes when we have two tensors that are identical in all but their free indices.

The simplest example is any column check $\delta_{,j}$ and row check $\delta_{i,}$ of a matrix - their contraction is one. To ensure commutativity, we need to guarantee that every pair of parity checks has a pair of $(2 \times \dots \times 2)$ tensors in their decomposition that share a free index in common, so that the overall parity is even. We can accomplish this by applying a permutation to the code which cyclically shifts the free indices in each $(2 \times \dots \times 2)$ tensor factor. Let $\sigma_a$ denote the $(a-1)$-fold cyclic shift of an $r$-element sequence. Then, we apply the permutation:

$$\begin{aligned} &\delta_{s_1,\dots,\hat{i_\ell},\dots,s_n} \otimes \left( \bigotimes_{k=1}^{r} \delta_{b_{k_1},\dots,\hat{i_\ell},\dots,b_{k_r}} \right) \mapsto \\ &\delta_{s_1,\dots,\hat{i_\ell},\dots,s_n} \otimes \left( \bigotimes_{k=1}^{r} \delta_{\sigma_k(b_{k_1},\dots,\hat{i_\ell},\dots,b_{k_r})} \right). \end{aligned}$$

Note that this does not change the code parameters, but does guarantee that in at least one of the subsystems, the free indices of the $Z$-type and $X$-type checks will be the same. Consequently, assigning the $Z$-stabilizers according to the parity checks of this code will yield a commuting set of stabilizers with the same distance. In the simple case of $r = 2$, this corresponds to transposing the last subsystem. For $r = 3$, this corresponds to cyclically permuting rows to columns to depths in the penultimate subsystem, and rows to depths to columns in the last subsystem.

Because adding the $Z$-stabilizers doubles the number of constraints, we obtain an $[[\prod_{i=1}^{r} n_i, 2\prod_{i=1}^{r} (n_i - 1) - \prod_{i=1}^{r} n_i, 2^r]]$ CSS code family that we call quantum multi-dimensional parity check codes (QPCCs). Note that each data qubit participates in two stabilizers per index, and so the total qubit degree is $2r$, while the maximum size stabilizer is $\max_i n_i$. In particular, the former constraint yields a matchable code in two dimensions or less.

We gave evidence that concatenating into 1D QPCCs reduced the number of physical qubits required for surface code storage by 1/2, and further by 2/3 when concatenating into 2D QPCCs. Given this trend, it is natural to ask: is

there any promise in continuing to concatenate into higher-dimensional QPCCs? They may prove harder to lay out, but by boosting the distance of smaller surface codes with higher distance high-rate outer codes, we can in principle increase the rate of logical qubits per physical qubit. However, we eventually hit diminishing returns as the complexity of the logical parity check circuitry increases.

Note also that one of the main advantages of our approach is the high rate of the outer code we concatenate into. For any fixed dimension, the rate of a QPCC approaches 1 as $n \to \infty$, as the number of constraints scale with the boundary of the array while the encoded degrees of freedom scale with the volume. However, lower dimensional codes achieve higher rates at much lower $n$, and in practice, this is an important consideration for integrating these memories into a fault-tolerant computation. Restricting to cube-like QPCCs with parameters $[[n^r, 2(n-1)^r - n^r, 2^r]]$, we observe that prohibitively large code sizes are required to achieve e.g. a 75% rate using higher-dimensional codes: for $r = 1$, we require $n = 8$; for $r = 2$, $n = 256$; for $r = 3$, $n = 13824$; and for $r = 4$, $n = 1048576$. This suggests that going beyond 2D QPCCs might be prohibitively expensive.


[1] Craig Gidney, Michael Newman, Austin Fowler, and Michael Broughton. A fault-tolerant honeycomb memory. *Quantum*, 5:605, 2021.

[2] Sergey Bravyi, Andrew W Cross, Jay M Gambetta, Dmitri Maslov, Patrick Rall, and Theodore J Yoder. High-threshold and low-overhead fault-tolerant quantum memory. *Nature*, 627(8005):778–782, 2024.

[3] Craig Gidney and Cody Jones. New circuits and an open source decoder for the color code. *arXiv preprint arXiv:2312.08813*, 2023.

[4] Craig Gidney. Inplace access to the surface code y basis. *Quantum*, 8:1310, 2024.
